# Altered activity of pain processing brain regions in association with hip osteoarthritis

**DOI:** 10.1038/s41598-022-06499-8

**Published:** 2022-02-18

**Authors:** P. Railton, A. J. Delaney, B. G. Goodyear, J. Matyas, S. Lama, G. R. Sutherland, J. N. Powell

**Affiliations:** 1 School of Biomedical Sciences, Charles Sturt University, Orange, Australia; 2 School of Dentistry and Medical Science, Charles Sturt University, Orange, Australia; 3 Cumming School of Medicine, Calgary, AB Canada; 4 Departments of Radiology, Clinical Neurosciences and Psychiatry, Hotchkiss Brain Institute, University of Calgary, Calgary, AB Canada; 5 Clinical Neurosciences & Hotchkiss Brain Institute, University of Calgary, Calgary, AB Canada

**Keywords:** Neuroscience, Anatomy, Pathogenesis

## Abstract

Hip osteoarthritis (OA) is characterized by chronic pain, but there remains a mismatch between symptoms and radiological findings. Recently, brain connectivity has been implicated in the modulation of chronic peripheral pain, however its association with perceived pain in hip OA is not understood. We used resting-state functional magnetic resonance imaging (fMRI) to examine functional connectivity associated with pain in hip OA patients. Thirty participants with hip OA and 10 non-OA controls were recruited. Using the visual analogue scale (VAS), pain scores were obtained before and after performing a painful hip activity. All participants underwent 3.0 T resting-state fMRI, and functional connectivity of brain regions associated with pain was determined and compared between participants, and before and after hip activity. Relative to controls, functional connectivity between the secondary somatosensory cortex and left posterior insula was increased, and functional connectivity between the bilateral posterior insula and motor cortices was significantly decreased in hip OA participants. In response to painful hip activity, functional connectivity increased between the thalamus, periaqueductal grey matter and brainstem. Functional connections between brain regions associated with pain are altered in hip OA patients, and several connections are modulated by performing painful activity. Unique lateralization of left posterior insula and linked brain functional connectivity patterns allows assessment of pain perception in hip OA providing an unbiased method to evaluate pain perception and pain modulation strategies.

## Introduction

The lack of association between radiological indicators of hip osteoarthritis (OA) and self-reported pain by individual patients^1–4^ emphasizes the long recognized, but poorly understood mismatch between symptoms and radiological signs of OA. This places limits on how we effectively treat the perceived pain associated with chronic hip OA. Whereas the hallmark signs of joint pathology—degradation and inflammation—are in the periphery, it is unclear why joint pain progresses from local, sharp and episodic, to widespread, dull and persistent. Several studies suggest that pathological changes, evident within the joint afferent neurons^5,6^, spinal cord^7^, and brain circuits^8,9^, are distinct features of long-standing OA. Thus, there is increasing recognition that mechanisms within the central nervous system (CNS) play a significant role in the modulation and perception of pain^10^. Indeed, central sensitization (typified by CNS neuroinflammation and neuroplasticity) characterizes neuropathic-like pain in OA patients^11–16^. In one study, pain was reduced and recovery was improved after total knee arthroplasty when patients were treated with the CNS-acting drug, duloxetine, a serotonin–norepinephrine reuptake inhibitor^17^. Hence, there is a need to better understand the impact of OA on the CNS such that patient-specific therapy can be tailored.

Functional magnetic resonance imaging (fMRI) has demonstrated changes within brain circuits involved in the perception of pain^18^.These studies using fMRI report increased activity within the thalamus, posterior and anterior insula, secondary somatosensory cortex, anterior cingulate cortex and periacqueductal grey matter. Wager called this a *pain signature* within the brain^19^. Other investigations using fMRI in patients with chronic pain have shown multiple changes, i.e., distinct signatures, in a number of brain regions^20^. In patients with hand OA, increased activation of the thalamus, anterior cingulate, frontal and somatosensory cortices was observed, relative to controls, when subjects were asked to perform a hand movement task^21^. Specific changes within the brain circuitry of knee OA patients have been reported to involve the periaqueductal grey matter, ventral tegmental area, and bilateral medial orbital prefrontal cortex^22^. Most recently, a resting-state fMRI study of knee OA patients following arthroplasty demonstrated changes in the temporal synchrony between the rostal anterior cingulate cortex and rostral ventromedial medulla^23^ (the degree of synchrony is thought to indicate the strength of the functional connection, and is called connectivity).

With regards to hip OA patients, alterations in periacqeductal gray matter activity in response to skin stimulation have been reported^11^. Interestingly, a volumetric brain MRI study of hip OA patients demonstrated evidence of reversal of thalamic atrophy following total joint arthroplasty^24^, suggesting that CNS changes may be reversible following the removal of the (peripheral) stimulus of chronic joint pain.

Collectively, these studies demonstrate a clear involvement of the CNS in OA; however, the pain signatures vary depending on the condition and joint itself. Hence, there is a need to investigate the association between hip OA pain and CNS brain circuity, to determine the potential of CNS-targeted therapies to improve the quality of life of hip OA patients. The present study used resting-state fMRI to explore differences in functional connectivity between hip OA patients and control subjects. Further, changes in connectivity were examined in hip OA patients following an acute painful hip activity.

## Materials and methods

This study was approved by the Conjoint Health Research Ethics Board of the University of Calgary (REB14-2375), and written Informed consent was obtained from all participants. All methods were performed in accordance with the relevant guidelines and regulations. Thirty patients with hip OA requiring total hip replacement were recruited from the Alberta Hip and Knee Clinic, in Calgary, Alberta (17 males/13 females; mean age 56 ± 9.0 years; mean body mass index 28.0 ± 4.3). As determined by SF-36 questionnaire, collection of a Harris Hip Score and interview, patients were recruited for the study if they had no history of other chronic pain issues (i.e. back pain, or involvent from other joints), diabetes, neurological or psychiatric disorders, or were using antidepressants or marijuna. None of the patients used regularly prescribed medications for analgesia. Many had intermittently tried nonsteroidal anti-inflammatory agents and acetaminophen with no sustained benefit. The patients were symptomatic for greater than 1 year, many for multiple years before qualifying for total joint arthroplasty.Ten healthy participants (4 males/6 females; mean age 52.9 ± 6.5; mean body mass index 25.0 ± 4.6) were also recruited and similarly assessed.

For both OA patients and controls, blood pressure, heart rate and oxygen saturation levels were acquired prior to the MRI session. Baseline levels of perceived pain was assessed for hip OA patients using a visual analogue scale (VAS) for pain^25–29^ ranging from 0 to 10, where 0 indicates no pain and 10 indicates severe pain. All participants underwent MRI using a 3.0 T GE Discovery MR750 scanner equipped with a 12-channel receive-only phased array head coil (GE Healthcare, Waukesha, WI). The MRI session consisted of resting-state fMRI using a gradient-recalled echo, echo planar imaging sequence (repetition/echo time = 2500/30 ms, flip angle = 75°, 150 total volumes, 64 × 64 matrix, 3-mm isotropic voxels), during which participants were instructed to keep their eyes open and focussed on a fixation cross at the center of a projection screen inside the MRI scanner bore. Anatomical images for registration of the resting-state data were collected using a three-dimensional magnetization-prepared rapid gradient echo sequence (inversion/repetition/echo time = 550/8.2/3.2 ms, 0.8 × 0.8 × 1.3 mm voxels). Hip OA participants and controls were removed from the MRI scanner and completed 10 min of physical activity engaging the hip (stair climbing, squatting and stretches as tolerated) and were re-assessed for pain using the VAS and for blood pressure, heart rate and oxygen saturation levels. The participants were immediately placed back inside the MRI scanner and underwent a repeated resting-state fMRI scan. VAS scores were compared between baseline and post painful activity by a Wilcoxon Signed-Rank test.

Resting-state fMRI images underwent pre-processing, including skull stripping, motion correction using a six degrees-of-freedom (DOF) rigid body trasnsformation, intensity normalization of the entire 4D volume, slice-timing correction using sinc interpolatiom to account for timing difference of each slice within the TR, high-pass temporal filtering using a 0.001 Hz cutoff, and 6-mm Gaussian kernel spatial smoothing. These steps were performed using FSL image analysis software (http://www.fmrib.ox.ac.uk/fsl/). Using *MELODIC* (part of the FSL software library), Independent component analysis (ICA) was used to identify time-varying signals arising from artifact due to non-physiological noise, residual non-corrected head motion, and cardiac and respiratory pulsatility, which were subsequently removed by linear regression^30^. The total number of ICA components reviewed were automatically estimated using *MELODIC* (typically 40-60) of which 4-6 components are identified as artifact. To permit group comparisons, resting-state fMRI images were registered to anatomical images and subsequently to the Montreal Neurological Institute’s (MNI) standard brain template, using a 12 DOF linear registration model. All images were visually inspected to ensure alignment with the brain template was successful using well-established anatomical landmarks (i.e. motor strip, basal ganglia, cerebellum and insula).

A region-of-interest (ROI) approach was used to analyse the resting-state fMRI data. Specific ROIs were chosen as ‘seed’ regions, to determine their functional connectivity with the rest of the brain and how connectivity differed between subject groups and after physical activity involving the hip. The ROIs included the secondary somatosensory cortex (S2), anterior/posterior insulae and thalamus, as these regions have been shown to be associated with acute onset of pain^19^ and are easily discernible on anatomical MR images using standard brain MRI templates. For each participant and for each of the baseline and post physical activity resting-state fMRI datasets, the average time varying signal of each ROI was obtained using FSL’s command-line tools and were subsequently used in separate time series analyses to determine functional connectivity of the ROI, as implemented in FEAT v6.0 (part of FSL). Comparisons of functional connectivity of each ROI between hip OA and control subjects as well as between baseline and after painful hip activity were determined using a mixed-effects analysis in FEAT. Results were presented as brain maps of the Z-score of the difference in connectivity, corrected for multiple comparisons using a false-discovery rate (FDR) threshold of 0.05, corresponding to a cluster volume of greater than 322 voxels, as determined by *AlphaSim* (part of the AFNI software package, http://afni.nimh.nih.gov/afni)^31^, which uses simulations of a null distribution of Z-scores given the spatial smoothness of the images. Other than the MR data, all results are presented as mean ± SD. Comparison between groups was performed using Student’s t-test, with significance level set at p < 0.05.

## Results

**Figure 1:**
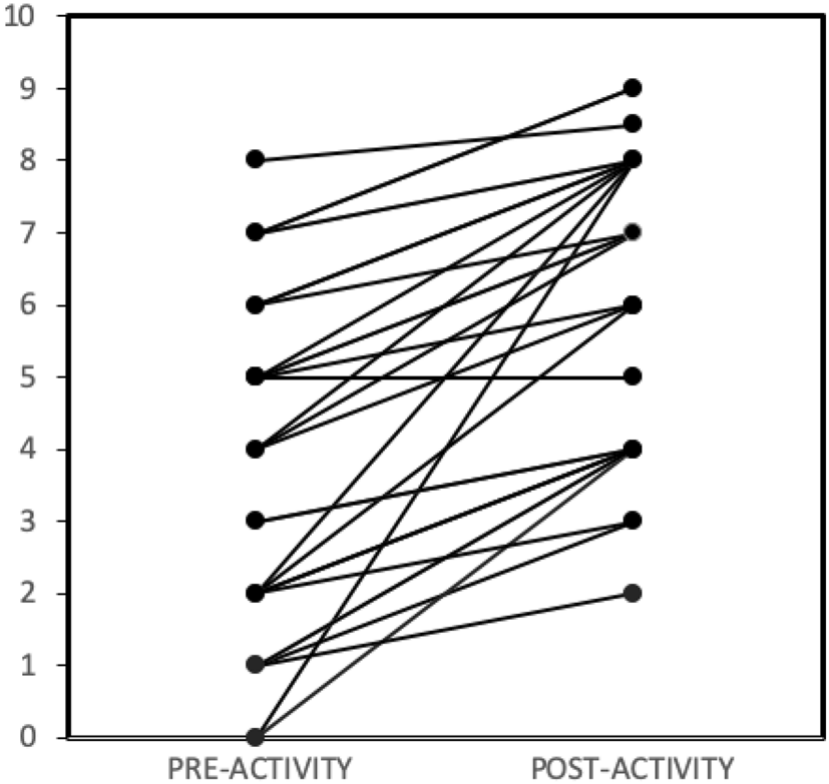
VAS scores for all hip OA patients (n = 30) before and after physical activity. Several patients exhibited the same pre- and post-activity scores, and hence their lines overlap.

The VAS scores of pain for hip OA patients at baseline and after physical activity are shown in Fig. 1. The scores increased significantly following physical activity of the hip (*p* < 0.001), confirming that the painful hip activity intervention was successful in reproducing the hip pain. Participants’ vital signs were monitored and the changes in the vital sign post exercise are noted in Table 1.

**Table 1:** Pre- and post-activity physiological measurements.

	PRE exercise	POST exercise
**OA (N = 30)**		
Systolic BP	130 ± 17	151 ± 16**
Diastolic BP	81 ± 12	88 ± 9**
HR	73 ± 11	84 ± 12**
O2 SAT	98 ± 1	96 ± 2**
**Controls (N = 10)**		
Systolic BP	123 ± 15	144 ± 17**
Diastolic BP	68 ± 10	82 ± 10*
HR	68 ± 9	93 ± 16**
O2 SAT	98 ± 0.7	97 ± 1

*p < 0.05.

**p < 0.01.

Functional connectivity based on seed regions and their activation comparing OA patients and controls is summarized in Table 2.

**Table 2:** Seed regions and connectivity comparisons between OA patients and controls pre activity.

Seed region	Increased connectivity	Decreased connectivity
**Baseline**		
Secondary somatosensory cortex	Left Posterior insula	
Posterior insula		Right temporal lobe hip motor/sensory cortex

The connectivity changes in the study patients before and after activity are shown in Table 3.

**Table 3:** Connectivity changes in study patients pre and post activity.

Seed region	Increased connectivity	Decreased connectivity
**Post-activity**		
Thalamus	Right brainstem Parahippocampal gyri Peri-aqueductal grey matter	
Anterior insula	Medial superior frontal gyri Anterior cingulate gyri	
Posterior insula	Brainstem Cerebellum Right putamen Right posterior insula	

**Figure 2:**
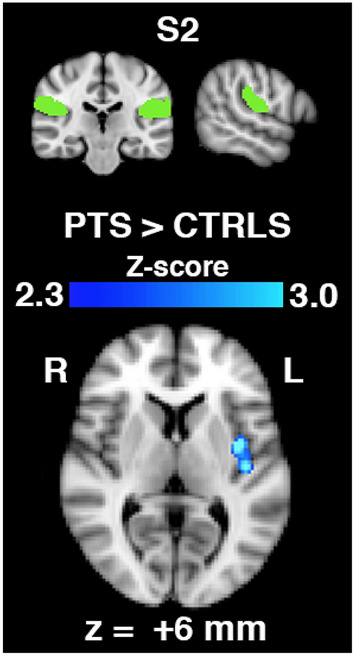
Brain regions (bottom, in blue-to-light blue) exhibiting greater connectivity with the secondary somatosensory cortex (S2) seed region (top, in green) in hip OA patients (n = 30) than in control subjects (n = 10), expressed as a statistical Z-score of the difference. Regions include only the left posterior insula (z-coordinate of shown brain slice corresponding to the MNI brain template.

Functional connectivity between the secondary somatosensory cortex (S2) and the left posterior insula was significantly greater in hip OA patients than in control subjects (Fig. 2). Because of the laterality of this finding, the analysis was repeated by separating left (n = 15) and right (n = 15) hip OA patients into groups; however, the results were unchanged.

**Figure 3:**
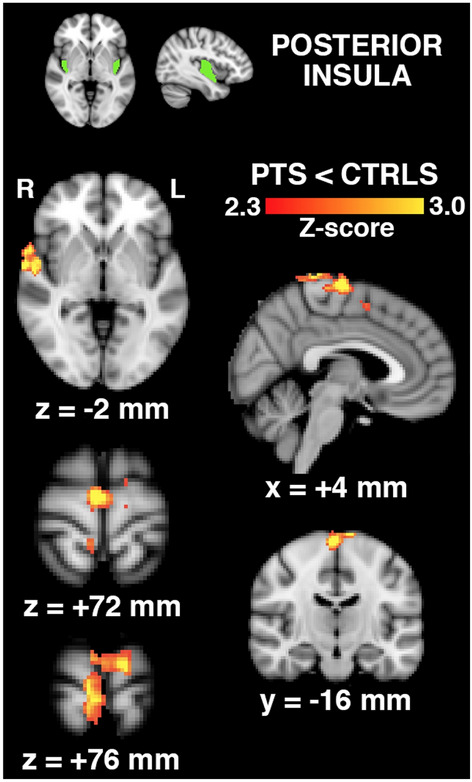
Brain regions (bottom, in red-to-yellow) exhibiting reduced connectivity with the bilateral posterior insula seed region (top, in green) in hip OA patients (n = 30) than in control subjects (n = 10), expressed as a statistical Z-score of the difference. Regions include the right temporal pole and regions of the motor and sensory cortices associated with hip movement and sensation (x,y,z-coordinates of shown brain slices correspond to the MNI brain template).

Brain regions that exhibited reduced connectivity with the bilateral posterior insula in hip OA patients relative to control subjects are shown in Fig. 3. Reduced connectivity was observed in the right temporal pole and regions of the primary motor and sensory cortices associated with movement and sensation of the hip. No significant findings were observed when comparing functional connectivity of the anterior insula or the thalamus between hip OA patients and control subjects.

The following 3 figures illustrate the changes in connectivity between seed regions in study patients only. The individual patient serves as their own control pre and post activity. No connectivity changes were noted in controls pre and post activity.

**Figure 4:**
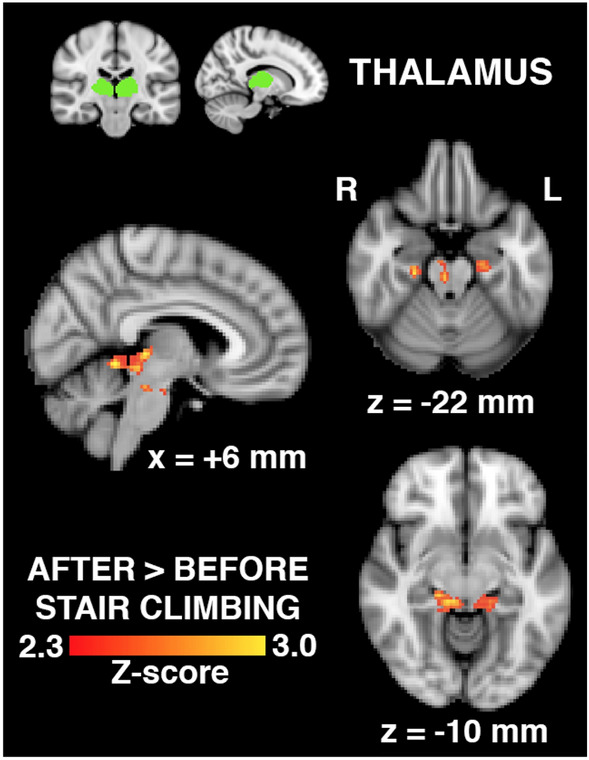
Brain regions (bottom, in red-to-yellow) exhibiting increased connectivity with the thalamus seed region (top, in green) in hip OA patients (n = 30) following physical activity involving the hip, expressed as a statistical Z-score of the difference. Regions include the right brainstem, left and right parahippocampal gyri, and the left and right periaqueductal grey (PAG) (x,z-coordinates of shown brain slices correspond to the MNI brain template).

Brain regions whose connectivity with the thalamus increased following physical activity engaging the hip are shown in Fig. 4. Regions included the right brainstem, left and right parahippocampal gyri, and the left and right periaqueductal grey (PAG) matter.

**Figure 5:**
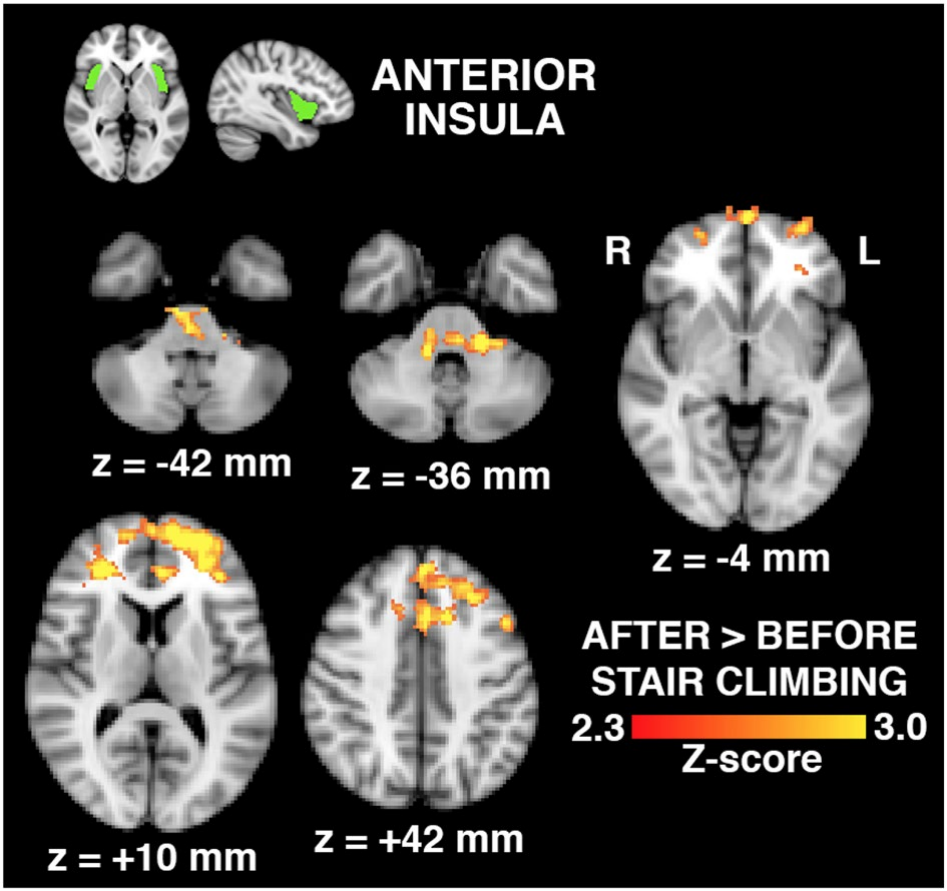
Brain regions (bottom, in red-to-yellow) exhibiting increased connectivity with the anterior insula seed region (top, in green) in hip OA patients (n = 30) following physical activity involving the hip, expressed as a statistical Z-score of the difference. Regions include the brainstem and regions within the left and right medial, superior and frontal gyri, as well as the anterior cingulate cortex (z-coordinates of shown brain slices correspond to the MNI brain template).

Brain regions whose connectivity with the anterior insula increased following physical activity engaging the hip are shown in Fig. 5. Regions included the brainstem and regions within the left and right medial superior frontal gyri, as well as the anterior cingulate cortex.

**Figure 6:**
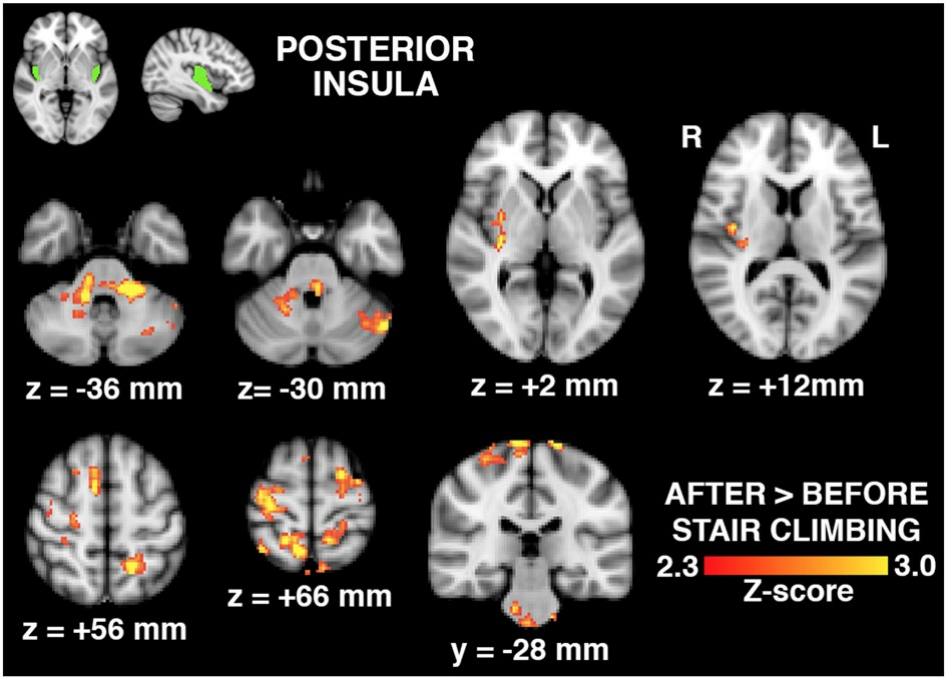
Brain regions (bottom, in red-to-yellow) exhibiting increased connectivity with the posterior insula seed region (top, in green) in hip OA patients (n = 30) following physical activity involving the hip, expressed as a statistical Z-score of the difference. Regions include the brainstem, bilateral cerebellum, right putamen and posterior insula, and regions of the motor and sensory cortices, including those associated with hip movement and sensation (y,z-coordinates of shown brain slices correspond to the MNI brain template).

Functional connectivity based on seed regions and their activation between OA patients and control group following activity have been summarized in Table 2. Brain regions whose connectivity with the posterior insula increased following physical activity engaging the hip are shown in Fig. 6. Regions included the brainstem, bilateral cerebellar hemispheres, right putamen and posterior insula, and regions of the motor and sensory cortices, including those associated with hip movement and sensation. No changes in functional connectivity with the anterior insula were observed for control subjects. No significant differences were observed between baseline and physical activity of the hip for connectivity with S2, for either the hip OA or control group.

## Discussion

The paradoxical disconnect between OA symptoms and radiologically apparent degenerative changes has long puzzled clinicians. As degeneration of articular cartilage is the hallmark pathological feature of OA, and cartilage has no direct nervous innervation, pain signalling in arthritis joints must emanate from surrounding tissues due to inflammation or otherwise^32,33^. As the natural history of joint degeneration typically occurs over a prolonged period, OA symptoms arise well after OA degeneration has progressed to involve periarticular tissues. Hence, there are few studies on the natural history of OA pain. Nevertheless, the present study sought to define the neuroplastic changes in the brain using resting-state fMRI in patients who were candidates for hip arthroplasty, both because all patients report chronic pain that worsens with provocative hip activities and hip arthroplasty is a treatment of choice that aims primarily to relieve the pain.

The present study demonstrates a difference in functional connectivity of controls and patients with severe osteoarthritis. We observed a unique signature of increased connectivity between the secondary somatosensory cortex and the left posterior insula. The posterior insula and its relationship to nociception has been well described^34^. Consistent with our observation, other investigators have also described functional connectivity of posterior insula to secondary motor and somatosensory cortices^35,36^. Furthermore, the connectivity of posterior insula with mid-cingulate cortex substantiates its role in pain sensation^37–39^. In our study, localization of activation to left posterior insula was somewhat surprising and novel. This could well relate to the left posterior insula’s predominant role in empathy and emotion with recognition of disgust^40^. The chronicity of OA could well be represented by continuous activation of the left posterior insular cortex, also observed for the first time, in our study. It would therefore be interesting to speculate as to whether or not, this lateralization to the left posterior insula would still be evident in early OA pain prior to coping behaviors and eventual development of central sensitization. The unilateral signal is present in OA patients, who, with one exception, were all right-hand dominant (noting that hip disease was equally split between the right and left sides). The majority, 88% of right handed people are left hemispheric dominant and 78% of left handed people are also left hemisphere dominant. It would be interesting to speculate if localization of pain to the left posterior insula would hold true for patients who are right hemisphere dominant, i.e. only ~ 7% of left handed people^41^.

Post-surgical fMRI studies could also lead to a fuller understanding of the potential reversibility of the observed imaging characteristics, particularly the circuitry involved in central sensitization associated with OA disease. Despite the cost related to MRI studies, the fMRI could be a worthwhile consideration as a tool for patients with chronic OA pain, including persistent post-surgical pain. The present study revealed that resting-state fMRI defines an unusual signature of brain connectivity characteristic of chronic OA hip pain at baseline.

Following acute pain brought on by painful hip activities, we observed patterns of altered interconnectivity of multiple brain regions sometimes referred to as the pain matrix^42^. Our findings are similar to what has been reported for brain activation following acute pain and include nociceptive, emotional and motivational circuitry^19,43–59^. When pain is acute and severe, brain regions involved in emotion, cognition and motivation are predominantly activated^60^.

While we present novel observation, the study does rely on a few assumptions and limitations. An important limitation is that all but one patients were right handed. In addition, the VAS methodology is based on subjective self-reporting by patients. While known to be variable, this methodology is widely used and has been shown to have validity for studies of OA pain^49^. As the same VAS was used repeatedly on each patient, before and after painful hip activities, each patient served as their own comparator for this measure of clinical hip pain. The documentation of brain connectivity afforded by the resting state fMRI analyses used here is based on an unbiased statistical methodology. The choice of predetermined ROIs permitted an hypothesis-based analysis; however, there may be other brain regions involved that are not revealed using our approach.The connectivity results presented here need to be considered in two phases: (a) connectivity differences between OA and control patients, and (b) connectivity differences in OA patients before and after provocative hip tests. Presumably, connectivity differences at baseline between OA and controls reflect neuroplastic changes that occur in response to chronic OA hip pain, which typically takes months or years to develop, conceivably in a timeframe that corresponds in some way with development of hip OA pathology. In contrast, changes from baseline connectivity detected in OA patients after provocative hip tests infers activation of pre-existing neural connections in the brain rather than structural neuroplastic changes. This later type of brain activation is analogous (but not homologous) to brain activation in BOLD fMRI images in response to a noxious stimulus (i.e., a paradigm). While these two connectivity signals are not mechanistically congruent nor directly comparable, they represent valid and intriguing brain signals corresponding to hip OA pain. As such, this resting-state fMRI approach would seem to be advantageous for evaluating both spontaneous (baseline) and evoked pain that in turn assess two important aspects of pain-circuit connectivity: development and activation.

One final limitation that can be recognized is that this assessment is only measuring functional connectivity and has not assessed structural changes in the brain. Baliki and coauthors have shown that different clinical conditions have different patterns of structural change in the brain, for example Chronic low back pain showed reduced whole-brain gray matter volume, whereas 2 other chronic pain conditions did not, Chronic Regional Pain Syndrome and Knee OA^61^. Future work should examine this component of change as well.

## Conclusion

In summary, the resting-state fMRI of patients with hip OA relative to controls demonstrated greater connectivity between secondary somatosensory cortex and the left posterior insula. Connectivity was reduced between the posterior insula and the hip area of the primary motor and sensory cortices. We believe that this is a resting-state fMRI signature for OA of the hip with chronic pain, and indeed may hold an important clue in understanding pain pathways in patients with chronic OA of the hip. Similar to other reports, we also demonstrated connectivity changes after painful hip activity.
